# Acute kidney injury: an acceptable risk of treatment with renin-angiotensin system blockade in primary care?

**DOI:** 10.1186/s40697-015-0044-y

**Published:** 2015-04-09

**Authors:** Michael Bedford, Christopher KT Farmer, Jean Irving, Paul E Stevens

**Affiliations:** Kent Kidney Research Group, East Kent Hospitals University NHS Foundation Trust, Kent and Canterbury Hospital, Ethelbert Road, CT1 3NG Canterbury, Kent

**Keywords:** Acute kidney injury, Renin-angiotensin system blockade, System for Early Identification of Kidney Disease (SEIK)

## Abstract

**Background:**

Use of renin-angiotensin system (RAS) blockade has become increasingly widespread driven by evidence-based guidance. There is concern about the role of these agents in the genesis of avoidable acute kidney injury (AKI).

**Objectives:**

To investigate the association between AKI and use of RAS blockade.

**Design:**

Multilevel hierarchical analysis of a large cohort of patients registered with UK general practitioners.

**Setting:**

Primary care practices in East and West Kent, United Kingdom.

**Patients:**

244,715 patients from 27 practices.

**Measurements:**

Demographic, clinical, biochemical and prescription data.

**Methods:**

Analyses of data acquired between 02/3/2004 and 17/04/2012 using multilevel logistic regression to determine the relationship between AKI and use of RAS blockade; further analysed by indication for treatment with RAS blockade.

**Results:**

Sufficient serum creatinine data were available to define AKI in 63,735 patients with 208,275 blood test instances. In 95,569 instances the patient was prescribed a RAS antagonist of which 5.4% fulfilled criteria for AKI. The unadjusted odds ratio (OR) for AKI in those prescribed RAS blockade was 1.93 (1.81-2.06, 95%CI) falling to 1.11 (1.02-1.20, 95%CI) when adjusted for age, gender, co-morbidity, GFR category, proteinuria, systolic blood pressure and diuretic therapy. In patients with an evidence-based indication there was no difference in absolute risk of AKI. However, prescription of RAS blockade in the absence of indication appeared to be associated with greater risk of AKI.

When analysis was repeated with AKIN2/AKIN3 as the outcome, although risk of AKI remained significant when unadjusted (OR 1.73, 95%CI 1.42-2.11, p<0.001), after full adjustment there was no increased risk (OR 0.83, 95%CI 0.63-1.09) in those taking RAS antagonists. However, when analysed by indication AKIN2/AKIN3 was significantly more likely in those prescribed RAS antagonists without indication (OR 2.04, 95%CI 1.41-2.94, p<0.001).

**Limitations:**

Observational database study. No information concerning hospitalisation. Prescribing assumptions and potential inaccurate coding. Potential survival bias; patients surviving longer will contribute more data.

**Conclusions:**

Use of RAS antagonists increased the risk of AKI, independent of common confounding variables. After correction for confounders the risk fell away and became non-significant for moderate and severe AKI. However, where there was no evidence-based indication for RAS antagonists the risk of AKI, whether mild, moderate or severe, remained greater.

## Abrégé

**Contexte:**

Vu l’abondance de données probantes en la matière, le recours aux inhibiteurs du système rénine-angiotensine-aldostérone (SRAA) est de plus en plus répandu. Il existe certaines préoccupations quant au rôle de ces agents dans la genèse de l’insuffisance rénale aiguë (IRA) évitable.

**Objectif de l’étude:**

Examiner, au sein d’une cohorte en soins de santé primaires, la présence de liens entre l’IRA et l’utilisation d’inhibiteurs du SRAA.

**Type d’étude:**

Une analyse hiérarchique multiniveaux d’une vaste cohorte de patients suivis par des médecins généralistes du Royaume-Uni.

**Contexte:**

Cliniques de soins de santé primaires situées dans l’est et l’ouest du comté du Kent, au Royaume-Uni.

**Patients:**

Les données ont été recueillies auprès d’une cohorte de 244 715 patients en soins primaires, provenant de 27 cliniques de soins primaires dans l’est et l’ouest du comté du Kent.

**Mesures:**

Données démographiques, cliniques, biochimiques et issues d’ordonnances.

**Méthodes:**

L’analyse des données recueillies entre le 2004/03/02 et le 2012/04/17 a été effectuée par régression logistique multiniveaux afin de déterminer la relation entre l’IRA et l’utilisation d’inhibiteurs du SRAA, et ensuite par indication de traitement avec des inhibiteurs du SRAA.

**Résultats:**

Une quantité suffisante de données relatives à la créatininémie était disponible pour évaluer l’IRA chez 63 735 patients, qui avaient eu au total 208 275 prélèvements sanguins. Chez 95 569 sujets, un inhibiteur du SRAA a été prescrit, et 5,4% (5 194) de ces derniers ont eu un épisode d’IRA. Chez les patientsrecevant un traitement fondé sur des indications probantes, 5,8% (4473 sur 76 517) ont eu un épisode d’IRA. Le risque relatif non ajusté (RR) d’IRA associé à l’utilisation d’un inhibiteur du SRAA était de 1,93 (1,81-2,06, 95% IC), diminuant à 1,11 (1,02-1,20, 95% IC) lorsqu’ajusté pour l’ âge, le sexe, la comorbidité, la catégorie de débit de filtration glomérulaire, la protéinurie, la pression artérielle systolique et le traitement diurétique. Chez les patients recevant un traitement par inhibiteurs du SRAA fondé sur des indications probantes, il n’y avait aucune différence de risque absolu d’IRA. Par contre, il semblait y avoir un lien entre la prescription d’inhibiteurs du SRAA en l’absence d’indications probantes et un risque accru d’IRA. Lorsque l’analyse a été répétée avec l’AKIN2/AKIN3 comme critère de jugement, le risque d’IRA associé à l’utilisation d’un inhibiteur du SRAA restait significatif dans le modèle non ajusté (RR 1,73, 95% IC 1,42-2,11, p < 0,001), mais aucune augmentation de risque n’a été observée après ajustement (RR 0,83, 95% IC 0,63-1,09). Par contre, le risque d’AKIN2/AKIN3 lié à l’utilisation d’un inhibiteur du SRAA était significativement plus élevée chez les patients qui recevaient ces agents sans indications probantes (RR 2,04, 95% IC, 1,41-2,94, p < 0,001).

**Limites de l’étude:**

Étude par observation de données prises dans des cliniques de soins primaires. Aucune information d’hospitalisation disponible (base de données de soins primaires). Interprétation des prescriptions et possibilité de codes erronés. Biais de temps d’immortalité possible : les patients qui vivent plus longtemps contribuent davantage à l’analyse par les prélèvements sanguins.

**Conclusions:**

Notre analyse montre que l’utilisation d’inhibiteurs du SRAA augmente le risque d’IRA. Le risque est indépendant de diverses variables de confusion, dont l’âge, la mesure de base de la fonction rénale, la présence de comorbidité pertinente et la pression artérielle systolique. Après correction pour les variables confusionnelles, le risque diminuait toujours : il devenait non significatif pour l’IRA modérée et sévère. Par contre, le risque d’IRA légere, modérée ou sévère demeurait élevé lorsque l’utilisation d’inhibiteurs du SRAA ne s’appuyait sur aucune indication probante.

Renin angiotensin system blockade is known to be associated with acute kidney injury. This is the first study to examine this association by evidence-based indication. Although renin angiotensin system blockade increases the risk of acute kidney injury overall, in those with an evidence-based indication the majority of the effect is explained by underlying co-morbidity. In people with no evidence-based indication prescription of renin angiotensin blockade is an independent predictor of acute kidney injury.

## Background

The use of renin-angiotensin system (RAS) blockade in the form of angiotensin converting enzyme inhibitors (ACEIs), angiotensin receptor blockers (ARBs) and more recently direct renin inhibitors (DRIs) is now widespread. These agents are effective in lowering of blood pressure, reducing proteinuria and amelioration of chronic kidney disease (CKD) progression [1-3]. Evidence supporting beneficial effects in proteinuric diabetic and non-diabetic kidney disease has informed clinical practice guideline recommendations in both CKD and diabetes [4-6]. Evidence for their benefit in ischaemic heart disease and heart failure has also informed guideline recommendations in the general population [7-10] such that treatment with RAS antagonists has clearly defined high quality evidence-based indications (well-designed, well-executed randomised controlled trials (RCTs) or well-conducted meta-analyses of such studies) in the following patient population settings:

1. proteinuria (albumin:creatinine ratio [ACR] >70 mg/mmol)
2. hypertension and proteinuria (ACR > 30 mg/mmol)
3. diabetes and proteinuria (ACR > 3 mg/mmol)
4. chronic heart failure
5. post acute myocardial infarction

In addition hypertension guidance recommends use of RAS antagonists in those with hypertension and age <55 years or resistant hypertension at any age [8] and in those aged ≥18 years of age with hypertension and CKD [10] (RCTs with minor limitations, well-designed, well-executed non–randomised controlled studies and well-designed, well-executed observational studies or well-conducted meta-analyses of such studies).

Outside these indications there is no evidence to support the choice of RAS antagonists over other classes of anti-hypertensive agent in the management of hypertension, with or without CKD. The majority of patients with CKD will not progress to ESRD and these patients are predominantly managed by primary care in the community. In England during 2012 prescriptions for ACEIs, ARBs and DRIs accounted for 6.0 percent of all prescription items [11]. Not all of these prescriptions will be for evidence-based indications and this widespread use of RAS antagonists has raised questions about possible harm without additional benefit, particularly in the elderly [12].

Despite these concerns over the safety of RAS antagonism, in particular in relation to AKI we do not know the level of risk of AKI associated with the routine prescription of these agents in primary care.

The aim of this study was to examine the relationship between prescription of RAS antagonists and development of AKI in the community.

## Methods

We performed a multilevel hierarchical analysis of a large cohort of patients registered with UK general practitioners.

Data were extracted from the System for Early Identification of Kidney Disease (SEIK) database. SEIK is a computerized decision support system developed to assist in the management of CKD. The system extracts anonymised demographic, clinical, biochemical and prescription data from primary care systems. Reports aiding and advising on the management of CKD generated using an automated decision tree matrix and several computer algorithms based on NICE guidance [4-6,8] are then returned to participating practices. For this study data were drawn from 27 GP practices across East and West Kent in the UK. Patients with GFR < 15 ml/min/1.73 m^2^ or on renal replacement therapy were excluded.

In initial analyses it was evident that a large proportion of patients may switch between treatment with and without RAS antagonists over time, and therefore comparing outcomes in terms of episodes of AKI between these as 2 distinct groups was not viable. We therefore chose to analyse the data at the serum creatinine blood test level. For each patient we extracted all recorded serum creatinine estimations between 02/3/2004 and 17/04/2012. Each serum creatinine then became a data point “blood test instance” at which we extracted and defined the independent and outcome variables.

In performing the analysis at the blood test level there was then the inherent risk that several blood tests for an individual patient could represent the same episode of AKI. Therefore for a given episode of AKI the analysis algorithm excluded all blood results 30 days either side of the peak AKI result, unless a result within the 30 days no longer defined AKI. In this instance subsequent results were not thought to be part of that AKI episode, either prior to the AKI in the 30 days preceding the peak AKI result or demonstrating recovery in the 30 days post the peak AKI.

At each data point, “blood test instance”, we extracted or determined: age, gender, co-morbidity (including hypertension, diabetes, ischaemic heart disease, and heart failure), GFR category, proteinuria, blood pressure readings and prescription data including all anti-hypertensive agents and RAS antagonists. For the purposes of determining proteinuria indications for RAAS antagonists the highest proteinuria result for each patient was used. Proteinuria was categorised as per the KDIGO CKD Clinical Practice Guideline 2012 into three categories: “normal to mildly elevated”, ACR (or equivalent) <3 mg/mmol; “moderately elevated”, ACR (or equivalent) 3–30 mg/mmol; and “severely elevated”, ACR (or equivalent) >30 mg/mmol [13]. There is variance in prescription of anti-hypertensives including RAS antagonists across primary care in terms of the length of prescription given to patients, and also in the coding of these prescriptions. In some practices the dose prescribed and the number of tablets prescribed is coded, however in others only the tablet strength is coded. We therefore made the assumption that if the last prescription date was within 70 days of the “blood test instance”, then the patient was still receiving the medication at that time, this was on the basis that the majority of patients receive a 2 month (60 day) supply of medication. At each “blood test instance” we also defined whether or not a patient had an evidence-based indication for treatment with RAS antagonists as described in the introduction.

In this study the outcome variable of interest was AKI in primary care. AKI was defined by the acute kidney injury network (AKIN) creatinine criteria [14] but using the lowest SCr in the 12 months prior to the date of the peak AKI result as the reference after the method of Lafrance et al [15]. Finally we analysed the association between ACE/ARB and AKIN2/3.

This work was supported by the East Kent Hospitals Charity and approved by East Kent Hospitals University NHS Foundation Trust R&D Department, R&D ref: 2010/RENAL/09.

### Statistical methods

The primary aim of this analysis was to examine the association between patients taking RAS antagonists and experiencing episodes of AKI. In the analyses AKI was considered primarily as a binary variable, present or absent (ie AKI or no AKI and AKIN2/AKIN3 or no AKI/AKIN1). A feature of the data was that there were multiple measurements from some patients and as a result of this it was unlikely that the outcome values were all independent of each other. It was likely that outcomes for the same patient at different time periods were more similar than from different patients. Therefore it was necessary to account for this in the data analysis. Due to the binary nature of the outcome, and the lack of independence of the data, the analyses were performed using multilevel logistic regression. Two-level multilevel models were used with individual measurements nested within patients.

The relationship between RAS antagonists and AKI could potentially be confounded by various other parameters. Therefore, the relationships between the two key variables were adjusted for several pre-determined factors. Variables considered as potentially confounding were: age, sex, hypertension, diabetes, ischaemic heart disease (IHD), heart failure, GFR, proteinuria, systolic blood pressure and diuretic usage. The status of each of these was updated at the time of every blood test.

GFR category was used in preference to the baseline GFR value, as there were several particularly large GFR values, which might have been influential in the analyses. The GFR categories used followed the KDIGO CKD Clinical Practice Guideline 2012 classification of CKD [13]. A series of four models were examined, each considering the effects of RAS antagonists with different combinations of adjustments for other variables. Model 1 was unadjusted, model 2 adjusted for age and sex, model 3 for all variables apart from proteinuria and model 4 for all variables.

The first analysis assumed a constant effect of RAS antagonists for all patients. Subsequently all patients remained in the analysis, but the interaction between RAS antagonists and an evidence-based indication for their use was included in the analysis. This allowed the effects of RAS antagonists to vary for patients with and without an indication.

## Results

**Figure 1:**
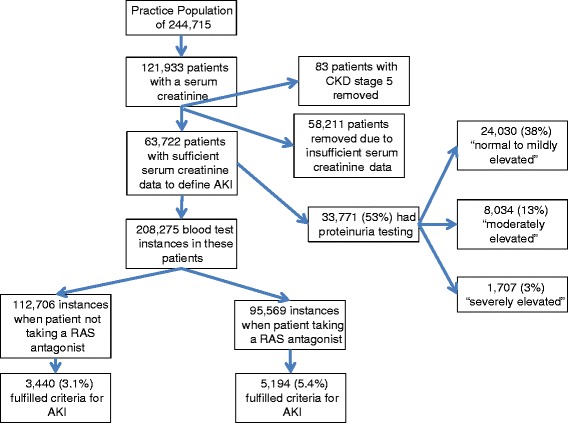
Study cohort and acute kidney injury (AKI) subdivided by RAS antagonist prescription and proteinuria testing.

There were 345,986 “blood test instances” from 121,933 patients in a practice population of 244,715. In 137,276 (39.7%) of the “blood test instances” no prior creatinine data were available and the presence or absence of AKI could not be assessed. The baseline characteristics of these subjects showed them to be significantly younger, with very little co-morbidity compared to those with baseline GFR data (Table 1). Only 5 percent had CKD and 21 percent hypertension, unsurprisingly they were prescribed significantly fewer medications and only 13 percent had an evidence-based indication for RAS blockade. 435 “blood test instances” from 83 patients were removed as the patient’s baseline GFR was <15 ml/min/1.73 m^2^. This left outcome data for 208,275 “blood test instances” from 63,722 patients. Table 1 demonstrates the population demographics of these 63,722 patients at baseline. In 112,706 of these instances the patient was not taking a RAS antagonist, 3.1% (3,440) of these instances fulfilled criteria for AKI. In 95,569 blood test instances the patient was taking a RAS antagonist, 5.4% (5,194) of these instances fulfilled criteria for AKI (Figure 1). Of the 63,722 patients: 27,970 (44%) also had proteinuria testing. Of these 22,552 (35%) had “normal to mildly elevated”, 4,473 (7%) had “Moderately elevated” and 945 (1.5%) had “Severely elevated” levels of proteinuria.

**Table 1:** **Population and baseline characteristics**

	**Analysed patients**			**No baseline GFR within the preceding year**
**Variable**	**Total population**	**No AKI in follow-up**	**AKI in follow-up**	
**Population**				
**Population in the analysis (%)**	63,722 (100)	58,904 (92.44)	4,818 (7.56)	49,695
**Average age (years)**	62.67	61.79	73.42	48.93
**Males (%)**	28,583 (44.86)	26,097 (44.30)	2,486 (51.60)	21,118 (42.50)
**Females (%)**	35,139 (55.14)	32,807 (55.70)	2,332 (48.40)	28,577 (57.50)
**GFR >60 ml/min/1.73 m** ^**2**^ **(%)**	50,283 (78.91)	48,135 (81.72)	2,148 (44.58)	47,175 (94.93)
**CKD Stage 3a (%)**	9,702 (15.23)	8,402 (14.26)	1,300 (26.98)	2,060 (4.15)
**CKD Stage 3b (%)**	3,019 (4.74)	2,038 (3.46)	981 (20.36)	399 (0.80)
**CKD Stage 4 (%)**	718 (1.13)	329 (0.56)	389 (8.07)	61 (0.13)
**CKD Total (%)**	13,439 (21.09)	10,769 (18.28)	2,670 (55.42)	2520 (5.07)
**Hypertension (%)**	38,912 (61.07)	34,962 (59.35)	3,950 (81.98)	10,454 (21.03)
**Diabetes (%)**	10,135 (15.91)	8,815 (14.97)	1,320 (27.40)	904 (1.81)
**Ischaemic Heart Disease (%)**	8,033 (12.61)	6,767 (11.49)	1,266 (26.28)	1163 (2.34)
**Heart Failure (%)**	916 (1.48)	628 (1.07)	288 (5.98)	63 (0.13)
**Had an indication for an ACEi/ARB (%)**	26,078 (40.92)	23,156 (39.31)	2,922 (60.65)	6,268 (12.61)
**Were on an ACEi/ARB (%)**	18,698 (71.70)	16,455 (71.06)	2,243 (76.76)	3,035 (6.12)
**Had no indication for an ACEi/ARB (%)**	37,644 (59.08)	35,748 (60.69)	1,896 (39.35)	43,427 (87.39)
**Were on an ACEi/ARB (%)**	5,236 (13.91)	4,751 (13.29)	485 (25.58)	1,095 (2.20)
**On a Thiazide Diuretic (%)**	12,628 (19.82)	11,384 (19.33)	1,244 (25.82)	2,796 (5.63)
**On another Diuretic (%)**	1,724 (2.71)	1,305 (2.22)	419 (8.70)	256 (0.52)
**On a Calcium Channel Blocker (%)**	17,744 (27.85)	15,785 (26.80)	1,959 (40.66)	2,488 (5.01)
**On a Beta Blocker (%)**	3,794 (5.95)	3,323 (5.64)	471 (9.78)	862 (1.73)
**On an Alpha Blocker (%)**	1,004 (1.58)	849 (1.44)	155 (3.22)	58 (0.12)
**On a Centrally Acting Agent (%)**	83 (0.13)	65 (0.11)	18 (0.37)	14 (0.03)
**Proteinuria (at study start):**				
**None recorded (%)**	35,752 (56.11)	33,504 (56.88)	2,248 (46.66)	35,014 (70.46)
**Normal to Mildly Elevated (%)**	22,552 (35.39)	20,945 (35.56)	1,607 (33.35)	13,342 (26.85)
**Moderately Elevated (%)**	4,473 (7.02)	3,732 (6.34)	741 (15.38)	1,065 (2.14)
**Severely Elevated (%)**	945 (1.48)	723 (1.23)	222 (4.61)	274 (0.55)

GFR (glomerular filtration rate), AKI (acute kidney injury), CKD (chronic kidney disease), ACEi (angiotensin converting enzyme inhibitor), ARB (angiotensin receptor blocker).

The majority of AKI was AKIN stage 1. Of the 3,440 instances where the patient was not taking a RAS antagonist, 3,194 had AKIN 1, 193 had AKIN 2, and 53 had AKIN 3. Of the 5,194 instances where the patient was taking a RAS antagonist, 4,881 had AKIN 1, 246 had AKIN 2, and 67 had AKIN 3.

To examine the possibility that a rise in serum creatinine associated with implementation of RAS antagonism led to a false assumption of AKI we also looked at the number of instances where a blood test occurred within 90 days of starting a RAS antagonist and at the percentage of those with AKIN1. In the first 90 days after initial RAS antagonist prescription only 194 instances fulfilled criteria for AKIN1 (4% of all AKIN1 in the study) representing only 2.5% of 7,765 blood test instances.

Table 2 shows the multilevel logistic regression results examining the association between RAS antagonists, and other variables, with AKI.

**Table 2:** **Multilevel logistic regression examining the association between renin angiotensin system antagonists and other variables with acute kidney injury**

**Variable**	**Category/term**	**Odds ratio (95% CI)**	**P-value**
**Model 1**			
ACE/ARB	No	1	**<0.001**
	Yes	1.93 (1.81, 2.06)	
**Model 2**			
ACE/ARB	No	1	**<0.001**
	Yes	1.69 (1.58, 1.81)	
Age ^(*)^	Linear term	0.41 (0.35, 0.48)	**<0.001**
	Quadratic term	1.12 (1.10, 1.13)	
Sex	Female	1	**<0.001**
	Male	1.70 (1.58, 1.83)	
**Model 3**			
ACE/ARB	No	1	**<0.001**
	Yes	1.17 (1.09, 1.25)	
Age ^(*)^	Linear term	0.48 (0.42, 0.56)	**<0.001**
	Quadratic term	1.08 (1.07, 1.09)	
Sex	Female	1	**<0.001**
	Male	1.61 (1.450, 1.72)	
Hypertension		1.30 (1.17, 1.44)	**<0.001**
Diabetes		1.47 (1.37, 1.58)	**<0.001**
IHD		1.24 (1.16, 1.35)	**0.001**
Heart Failure		2.29 (2.04, 2.56)	**<0.001**
Systolic BP < 100		2.32 (2.09, 2.58)	**<0.001**
CKD stage	1	1	**<0.001**
	2	1.90 (1.76, 2.04)	
	3	3.79 (3.46, 4.14)	
	4	6.79 (5.93, 7.77)	
Diuretic		1.42 (1.34, 1.51)	**<0.001**
**Model 4**			
ACE/ARB	No	1	**0.01**
	Yes	1.11 (1.02, 1.20)	
Age ^(*)^	Linear term	0.69 (0.55, 0.87)	**<0.001**
	Quadratic term	1.05 (1.03, 1.06)	
Sex	Female	1	**<0.001**
	Male	1.51 (1.40, 1.64)	
Hypertension		1.36 (1.18, 1.56)	**<0.001**
Diabetes		1.13 (1.04, 1.23)	**0.004**
IHD		1.28 (1.17, 1.40)	**<0.001**
Heart Failure		2.10 (1.85, 2.38)	**<0.001**
Systolic BP < 100		2.28 (2.01, 2.59)	**<0.001**
CKD stage	1	1	**<0.001**
	2	1.82 (1.67, 1.99)	
	3	3.40 (3.06, 3.77)	
	4	5.12 (4.38, 5.99)	
Diuretic		1.45 (1.35, 1.56)	**<0.001**
Proteinuria	None	1	**<0.001**
	Moderate	1.83 (1.69, 1.99)	
	Severe	3.27 (2.87, 3.72)	

(*) Odds ratios given for a 10-unit increase in the explanatory variable. Odds ratios describe the effect of all variables upon the outcome. For variables measured on a categorical scale, the odds ratios represent the odds of AKI in each category relative to a baseline category. For the continuous variables, the odds ratios represent the change in the odds of AKI for one-unit increase in that variable. A series of four models were examined, each considering the effects of RAS antagonists with different combinations of adjustments for other variables. Model 1 was unadjusted, model 2 was adjusted age and gender, model 3 for all variables apart from proteinuria and model 4 for all variables. CKD (chronic kidney disease), ACE (angiotensin converting enzyme inhibitor), ARB (angiotensin receptor blocker), IHD (ischaemic heart disease), BP (blood pressure).

**Figure 2:**
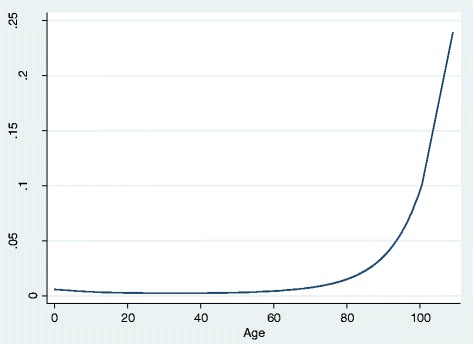
Relationship between age and the probability of AKI.

**Figure 3:**
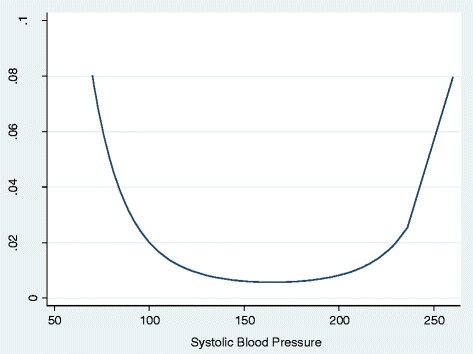
The relationship between systolic blood pressure and the probability of AKI.

The results for all four models suggested that treatment with RAS antagonists was significantly associated with an increased risk of AKI. The size of the effect decreased after adjustments for potential confounders falling from a 93% increased risk in the unadjusted model to 69% after adjustment for age and gender and to 11% in the fully adjusted model. All of the confounding variables examined were significantly associated with AKI. There was an increased risk of AKI for patients with hypertension, diabetes, ischaemic heart disease (IHD), heart failure, worsening severity of CKD, proteinuria and diuretic therapy. Males were at an increased risk relative to females. There was a non-linear relationship between age and AKI, and thus it is easier to view the results graphically (Figure 2), the results suggesting that for patients aged less than 60 years there was no strong relationship between age and risk of AKI. In those aged 60 and above the risk increased exponentially. There was a non-linear relationship between systolic blood pressure and AKI, and thus again it is easier to view the results graphically (Figure 3). Both hypotension and hypertension were associated with an increased risk of AKI.

Table 3 shows the multilevel logistic regression results examining the association between RAS antagonists, and other variables, this time with AKIN2/AKIN3 or noAKI/AKIN1. Only the first 2 models (unadjusted 73% increased risk, age and gender adjusted 62% increased risk) suggested that treatment with RAS antagonists was significantly associated AKIN2/AKIN3. After adjusting for the remaining variables (models 3 and 4), there was no statistically significant difference in the occurrence of AKIN2/3 status between those taking and not taking ACE/ARBs. In this analysis only hypertension, systolic blood pressure, use of diuretics and presence of proteinuria of the confounding variables were significantly associated with AKIN2/AKIN3.

**Table 3:** **Multilevel logistic regression examining the association between renin angiotensin system antagonists and other variables with AKIN2/AKIN3 compared with noAKI/AKIN1**

**Variable**	**Category/term**	**Odds ratio (95% CI)**	**P-value**
**Model 1**			
ACE/ARB	No	1	**<0.001**
	Yes	1.73 (1.41, 2.11)	
**Model 2**			
ACE/ARB	No	1	**<0.001**
	Yes	1.62 (1.31, 1.99)	
Age ^(*)^	Linear term	0.44 (0.30, 0.67)	**<0.001**
	Quadratic term	1.09 (1.06, 1.13)	
Sex	Female	1	0.21
	Male	1.15 (0.92, 1.43)	
**Model 3**			
ACE/ARB	No	1	0.85
	Yes	1.02 (0.81, 1.29)	
Age ^(*)^	Linear term	0.43 (0.27, 0.68)	**<0.001**
	Quadratic term	1.08 (1.04, 1.12)	
Sex	Female	1	0.34
	Male	1.12 (0.89, 1.40)	
Hypertension		1.87 (1.27, 2.74)	**0.001**
Diabetes		1.69 (1.33, 2.15)	**<0.001**
IHD		0.96 (0.73, 1.25)	0.75
Heart Failure		2.09 (1.44, 3.05)	**<0.001**
Systolic BP < 100		4.37 (3.18, 5.99)	**<0.001**
CKD stage	1	1	**<0.001**
	2	1.32 (1.01, 1.71)	
	3	1.78 (1.29, 2.45)	
	4	2.39 (1.47, 3.91)	
Diuretic		1.66 (1.34, 2.06)	**<0.001**
**Model 4**			
ACE/ARB	No	1	0.17
	Yes	0.83 (0.63, 1.09)	
Age ^(*)^	Linear term	0.47 (0.24, 0.93)	**<0.001**
	Quadratic term	1.07 (1.02, 1.12)	
Sex	Female	1	0.79
	Male	1.06 (0.81, 1.40)	
Hypertension		2.17 (1.29, 3.65)	**0.003**
Diabetes		1.32 (0.99, 1.74)	0.06
IHD		1.07 (0.78, 1.46)	0.69
Heart Failure		1.66 (1.07, 2.56)	**0.02**
Systolic BP < 100		4.34 (2.96, 6.36)	**<0.001**
CKD stage	1	1	0.47
	2	1.16 (0.85, 1.56)	
	3	1.31 (0.89, 1.92)	
	4	1.38 (0.76, 2.45)	
Diuretic		1.56 (1.20, 2.02)	**0.001**
Proteinuria	None	1	**<0.001**
	Moderate	1.62 (1.21, 2.17)	
	Severe	3.43 (2.22, 5.29)	

AKI (acute kidney injury), AKIN1 (acute kidney injury network stage 1), AKIN2 (acute kidney injury network stage 2), AKIN3 (acute kidney injury network stage 3), sCKD (chronic kidney disease), ACE (angiotensin converting enzyme inhibitor), ARB (angiotensin receptor blocker), IHD (ischaemic heart disease), BP (blood pressure).

(*) Odds ratios given for a 10-unit increase in the explanatory variable.

### By indication

The analysis was then repeated with at each “blood test instance” an assessment made of whether there was an evidence-based indication for the prescription of a RAS antagonist, other than simple hypertension. The exception was proteinuria where the highest proteinuria result was used. Table 4 summarises the association between indication, RAS antagonist prescription and AKI sub-divided by the two differing scenarios (no AKI versus AKI and no AKI/AKIN1 versus AKIN2/AKIN3).

**Table 4:** **The association between evidence-based indication, prescription of renin angiotensin system antagonist and acute kidney injury**

**Indication for ACE/ARB**	**ACE/ARB**	**No AKI**	**AKI**
		N (%)	N (%)
No	No	83,724 (97.8%)	1,846 (2.2%)
	Yes	18,331 (96.2%)	721 (3.8%)
Yes	No	25,542 (94.1%)	1,594 (5.9%)
	Yes	72,044 (94.2%)	4,473 (5.8%)
No AKI/AKIN1 versus AKIN2/AKIN3			
Indication for ACE/ARB	ACE/ARB	No AKI/AKIN1	AKIN2/AKIN3
		N (%)	N (%)
No	No	85,428 (99.83%)	142 (0.17%)
	Yes	18,989 (99.67%)	63 (0.33%)
Yes	No	27,032 (99.62%)	104 (0.38%)
	Yes	76,267 (99.67%)	250 (0.33%)

AKI (acute kidney injury), AKIN1 (acute kidney injury network stage 1), AKIN2 (acute kidney injury network stage 2), AKIN3 (acute kidney injury network stage 3), CKD (chronic kidney disease), ACE (angiotensin converting enzyme inhibitor), ARB (angiotensin receptor blocker), IHD (ischaemic heart disease), BP (blood pressure).

This summary suggests a greater effect of RAS antagonists on AKI for patients prescribed RAS antagonists with no evidence-based indication. If there was an indication for RAS antagonist prescription then there was no real difference in the risk of AKI. In the patients prescribed RAS antagonists without an evidence-based indication there appeared to be an increase in the risk of AKI.

The multilevel logistic regression was then repeated to examine the effects of RAS antagonists on AKI in the groups with and without an evidence-based indication (Table 5), using only model 1 (unadjusted) and model 2 (adjusted for age and sex) of the previous four models described above. Model 3 and model 4 were not used as the presence of co-morbidities such as diabetes, heart failure etc. and the presence of proteinuria would by definition give the patient an indication for RAS antagonist prescription and hence both these variables and indication could not be corrected for in the same model. Terms for the indication of RAS antagonists and also an interaction term between this variable and the actual occurrence of RAS antagonist prescription were included in the model.

**Table 5:** **Multilevel logistic regression examining the association between renin angiotensin system antagonists and acute kidney injury by evidence-based indication (model 1 shows the effects of renin angiotensin system antagonists with no adjustment, model 2 is adjusted for age and gender)**

**Model**	**Interaction p-value**	**Indication**	**Odds ratio (95% CI)**	**P-value**
No AKI versus AKI				
Model 1	<0.001	No	1.94 (1.72, 2.19)	**<0.001**
		Yes	1.14 (1.04, 1.24)	**0.004**
Model 2	0.003	No	1.52 (1.34, 1.72)	**<0.001**
		Yes	1.22 (1.12, 1.33)	**<0.001**
**No AKI/AKIN1 versus AKIN2/AKIN3**				
Model 1	<0.001	No	2.31 (1.61, 3.30)	**<0.001**
		Yes	0.98 (0.74, 1.30)	0.90
Model 2	0.005	No	2.04 (1.41, 2.94)	**<0.001**
		Yes	1.05 (0.79, 1.39)	0.73

AKI (acute kidney injury), AKIN1 (acute kidney injury network stage 1), AKIN2 (acute kidney injury network stage 2), AKIN3 (acute kidney injury network stage 3).

Analysis of the data in this was way suggested that there was a significant interaction between evidence-based indication and RAS antagonist use. In both models the risk of AKI was significantly higher with RAS antagonist use in both subgroups. However, the effects appeared greater in patients with no evidence-based indication.

## Discussion

Where there is an evidence-based indication for RAS antagonists over and above simple hypertension the literature suggests clear benefits in terms of reduction in all cause and cardiovascular mortality, progression of CKD and reduction in proteinuria [1-3]. Our study demonstrates an increased risk of AKI occurring in primary care in all patients prescribed RAS antagonists even after multiple adjustment for confounding risk factors, importantly including adjustment for systolic blood pressure. However, that risk becomes much lower in the fully adjusted model and when the analysis was repeated for moderate and severe AKI there was no increased risk associated with RAS antagonist prescription in the fully adjusted model. Furthermore, when analysed by evidence-based indication for RAS blockade, although there was no increased risk of AKI in those prescribed RAS antagonists with an indication, in patients prescribed RAS antagonists without an evidence-based indication the risk of AKI was significantly increased. This raises the question of whether or not risk outweighs benefit where there is no indication for RAS antagonist prescription over and above simple hypertension. We know from published data that all stages of AKI, even AKIN1, confer an increased risk of adverse outcome [15-23].

In high risk situations such as cardiac surgery the risk of AKI in those prescribed RAS antagonists preoperatively is significantly increased, by 27.6% in one study [24]. There are surprisingly few studies that have specifically addressed the risk of AKI in all patients prescribed RAS antagonists, and our study is the first to attempt to examine this by evidence-based indication. A recent ecological analysis suggested that up to 15% of the increase in AKI admissions in England over a 4-year time period was potentially attributable to increased prescribing of RAS antagonists but these findings were limited by the lack of patient level data including indication for prescribing and patient characteristics [25]. Lapi and colleagues examined the risk of AKI associated with the concurrent use of diuretics, RAS antagonists and non-steroidal anti-inflammatory drugs (NSAIDs) in a nested case–control study. They reported that the triple therapy combination consisting of diuretics RAS antagonists and NSAIDs was associated with an increased risk of AKI but that dual therapy combinations were not [26]. Harel et al. conducted a systematic review of published and unpublished RCTs that provided numerical data on adverse event outcomes, including AKI, when comparing monotherapy or combined treatment with different classes of RAS antagonists. The risk of AKI (defined as a serum creatinine concentration greater than 176.8 μmol/L) was no greater with combination therapy versus monotherapy [27].

Why is it that we find an increased risk of AKI in those prescribed RAS antagonists in the absence of an evidence-based indication, but not in those with an indication for prescription? It is likely that this relates to the relative contribution of confounding variables to risk of AKI. When we examined the risk of all AKI conferred by prescription of RAS antagonists that risk fell when adjusted for confounding variables and there was no increased risk of moderate to severe AKI after adjustment. We conjecture that because significant comorbidities such as systolic hypotension, heart failure and proteinuria are absent in those without an evidence-based indication for RAS antagonists prescription the contribution of RAS antagonism in such patients is that much more significant. There may also be a lower level of awareness and monitoring in those with fewer co-morbidities.

Our study has limitations. The study cohort is derived from the primary care population with recorded serum creatinine estimations. Although serum creatinine tests were recorded in 50 percent of the whole primary care population serum creatinine testing in primary care is not random. People with diabetes, hypertension and cardiovascular disease are over represented within our serum creatinine sample. In just under half of the population with serum creatinine estimations there were no baseline data to determine the risk of AKI and these patients could not be considered further. However, in the data analysed the absolute number of serum creatinine tests in those prescribed RAS antagonists was not dissimilar to the number in those not prescribed RAS antagonists. Furthermore those with no baseline data to determine risk of AKI were significantly younger with very little co-morbidity and only 5 percent had CKD. In this analysis we could not determine absolute risk of AKI and it is also important to note that the analysis only included blood tests from primary care, and therefore there is the possibility that we have not accounted for episodes of AKI that were managed in hospital and from which no blood tests were recorded in primary care. This is however a potential strength, as this excludes hospital acquired AKI and possible additional confounders. Another limitation is in the prescribing assumptions. Although primary care databases record the prescription of a drug the quantity given is often not available, and this may range for example from 1 – 3 months. We therefore made the assumption that if the last prescription date was within 70 days of the “blood test instance”, then the patient was still receiving the medication at that time. Although we were able to include diuretics in the analysis we were unable to accurately define the impact of non-steroidal anti-inflammatory combinations with other agents on the risk of AKI and therefore did not include this in the analysis.

Whilst there may be inaccuracies in database coding of co-morbidities individual patient level co-morbidity coding in primary care databases has been shown to be accurate, allowing correction for a number of known confounders in our analysis [28]. The introduction of the Quality Outcomes Framework, a pay for performance system in primary care in the United Kingdom, is likely to have further improved co-morbidity recording as targets for chronic disease management have been implemented [29].

This data set is also subject to survival bias in that people who live longer may contribute more tests to the analysis. Another potential source of bias was a misdiagnosis of AKIN1 purely as a result of change in serum creatinine following introduction of RAS antagonists. However, only 4% of AKIN1 occurred within 90 days of starting treatment with a RAS antagonist making this an unlikely source of significant bias.

This is the first study that identifies the risks associated with the indiscriminate use of RAS antagonists in a large general population cohort. For the first time we present data concerning the potential adverse effects of RAS antagonists in patients without a clear evidence-based indication for their use other than simple hypertension. Inclusion of all adults is a particular strength as older people are largely under-represented in randomised controlled trials of RAS antagonists and the incidence of AKI rises exponentially with age. A further strength of the study is the access to complete prescription data because primary care in England records all prescription data electronically.

Use of RAS antagonists independently predicted AKI in the multivariate analysis and it should be noted that in people with no evidence-based indication for treatment with RAS antagonists the risk of AKI was significantly increased. There will always be disease groups where the benefits of treatment with RAS antagonists clearly outweigh the risks, however we submit that treatment with these agents should be restricted to people in whom there is a clear evidence-based indication. Given the increasing incidence of AKI with increased age this is especially important in older people.

Strategies to mitigate the risk of AKI in people prescribed RAS antagonists should be encouraged, including regular monitoring of kidney function and the use of tablet holidays during intercurrent illness, especially that likely to involve intravascular volume depletion.

## Conclusion

In conclusion the use of RAS antagonists increased the risk of mild AKI in the community in this analysis and was independent of common confounding variables including age, baseline kidney function, gender, relevant co morbidities and systolic blood pressure. The risk of moderate to severe AKI was also increased by prescription of RAS antagonists but was no longer significant when fully adjusted for confounders. However, where there was no evidence-based indication for use of RAS antagonists the risk of mild, moderate and severe AKI remained significantly increased.
